# Clinical Prospects for Artificial Intelligence in Obstetrics and Gynecology

**DOI:** 10.31662/jmaj.2024-0197

**Published:** 2024-12-13

**Authors:** Kenbun Sone, Ayumi Taguchi, Yuichiro Miyamoto, Mayuyo Uchino-Mori, Takayuki Iriyama, Yasushi Hirota, Yutaka Osuga

**Affiliations:** 1 Department of Obstetrics and Gynecology, Faculty of Medicine, The University of Tokyo, Tokyo, Japan

**Keywords:** artificial intelligence, deep learning, obstetrics and gynecology

## Abstract

In recent years, artificial intelligence (AI) research in the medical field has been actively conducted owing to the evolution of algorithms, such as deep learning, and advances in hardware, such as graphics processing units, and some such medical devices have been used in clinics. AI research in obstetrics and gynecology has also increased. This review discusses the latest studies in each field. In the perinatal field, there are reports on cardiotocography, studies on the diagnosis of fetal abnormalities using ultrasound scans, and studies on placenta previa using magnetic resonance imaging (MRI). In the reproduction field, numerous studies have been conducted on the efficiency of assisted reproductive technology as well as selection of suitable oocyte and good embryos. As regards gynecologic cancers, there are many reports on diagnosis using MRI and prognosis prediction using histopathology in cervical cancer, diagnosis using hysteroscopy and prediction of molecular subtypes based on histopathology in endometrial cancer, and diagnosis using MRI and ultrasound as well as prediction of anticancer drug efficacy in ovarian cancer. However, concerns related to AI research include handling of personal information, lack of governing laws, and transparency. These must be addressed to facilitate advanced AI research.

## Introduction

The first AI boom occurred from the 1950s to the 1960s. Researchers believed they could develop programs that mimicked human intelligence, and the first AI systems emerged. During this time, search algorithms and inference engines were developed. However, the technology of the time had limitations in dealing with complex problems, and AI did not achieve the expected results. This led to the first AI winter in the 1970s. The second AI boom occurred in the 1980s when AI gained attention again. A major factor behind this was the development of expert systems. Expert systems used specialized knowledge in specific fields to solve problems and were applied in areas such as business diagnostics and decision-making support. However, this boom did not last long, as it became clear that expert systems could not handle the complexities of real-world problems. As a result, the second AI winter occurred in the early 1990s. After these booms and winters of artificial intelligence (AI), the third AI boom arrived, and AI has become an indispensable tool in our lives ^(1), (2)^. The reasons for the third AI boom are the growth of big data as well as the proliferation of the Internet and mobile devices, which have generated vast amounts of data and made rich datasets available for AI learning. The development of innovative algorithms, particularly in deep learning, such as convolutional neural network (CNN), has dramatically improved AI performance. Advances in hardware, such as graphics processing units, have made it possible to process large amounts of data and train deep-learning models faster. Thus, AI research in the medical field has been tremendous, and the number of research reports on these technologies has rapidly increased ^(3), (4)^.

Deep learning is effective for image recognition, and AI analyzes image data, such as radiography, computed tomography, and magnetic resonance imaging (MRI) scans, contributing to early detection and improved diagnostic accuracy ^(5)^. An increasing number of AI-powered medical devices have been approved overseas and in Japan. We herein review the latest AI research in obstetrics and gynecology.

## Obstetrics

### Cardiotocography

Cardiotocography (CTG) monitors fetal heart rate (FHR) and uterine contractions and is widely employed as a screening tool to determine fetal health during pregnancy and delivery. Therefore, numerous studies have been conducted on AI use related to CTG.

An important first step in the development of AI-based automation related to CTG is FHR detection. Metabolic acidosis-associated asphyxia is a common cause of fetal death. Zhong et al. collected 43,888 CTG data records of parturient women. After filtering the data, 2341 records were utilized. The AI model developed in this study, CTG-NET, performed well in the automated analysis of FHR ^(6)^. Imane et al. developed DeepCTG^Ⓡ^ 1.0, a model that can predict fetal acidosis using CTG data. It uses four features (minimum and maximum baseline FHR values and regions of acceleration and deceleration) extracted from 30-min CTG findings. The model was trained and evaluated using three different datasets, including UApH values. The results were relatively good, with an area under the curve (AUC) of 0.7-0.8 for predicting UApH (<7.05) ^(7)^. Gude developed an AI model to discriminate between late and variable decelerations. The algorithm developed in this study was employed to predict umbilical cord pH. The ensemble classification algorithm exhibited an 85% accuracy in predicting fetal acidosis based on the features extracted from the CTG data ^(8)^. Although AI studies on CTG exhibit accuracy, AI currently seems insufficient for practical use in CTG. Larger sample sizes seem necessary in future studies for improved outcomes.

### Ultrasound scan

In the perinatal field, several studies have reported the use of deep learning in fetal ultrasound. To apply machine learning to fetal ultrasound findings, it is important to accurately detect small structures, such as the ventricular septum, which dynamically changes in shape with each heartbeat and is difficult to detect using conventional segmentation methods. The color structure code (CSC) is characterized by the use of time-series and cross-sectional information. The ventricular septum was annotated using 421 normal fetal echocardiographic videos of 211 pregnant women who underwent fetal ultrasound screening. A comparison between CSC segmentation and conventional segmentation methods, DeepLab v3+ and U-net, showed that CSC performed better, with mean crossover union values of 0.0224, 0.1519, and 0.5543, respectively ^(9)^. Tang et al. developed a genetic disease-screening model using deep learning based on facial information from fetal ultrasound. They designed a two-stage ensemble learning model, Fgds-EL, based on ultrasound examinations and aimed to detect genetic diseases from 932 images. The genetic diseases addressed in this study were mainly trisomies 21, 13, and 18. This study yielded sensitivity and specificity of 0.92 and 0.97, respectively, which is comparable to that of advanced sonographers; therefore, this model can serve as a screening method for fetal genetic diseases in the future ^(10)^. Cystic hygroma has an incidence rate of 1 in 8,000 births and is associated with chromosomal abnormalities, such as trisomy 21 and Turner syndrome. Walker et al. developed a deep-learning algorithm for the diagnosis of cystic hygroma from fetal ultrasound images. The dataset included 129 cystic hygroma cases from 289 sagittal fetal ultrasound images and 160 normal nuchal translucency cases as controls. The overall model performed well, exhibiting an accuracy of 93% (95% confidence interval [CI]: 88%-98%), sensitivity of 92% (95% CI: 79%-100%), specificity of 94% (95% CI: 91%-96%), and area under the receiver operating standard curve of 0.94 (95% CI: 0.89-1.0) ^(11)^. Fetal ultrasound is a difficult field in the social implementation of AI medical devices as the fetus and its heart are moving targets. However, targeting organ- and disease-specific abnormalities rather than general fetal abnormalities could enable the use of AI medical devices in clinical practice.

### MRI

MRI may be performed during the perinatal period, although not as well as ultrasonography, depending on the disease. Shahedi et al. utilized a CNN for the simultaneous segmentation of the uterine cavity and placenta in MRI. The network was first trained on MRI of 181 patients: 157 for training and 24 for validation. The segmentation performance of the algorithm was evaluated using MRI of 60 additional patients not involved in the training, and the average Dice similarity coefficients for the uterine cavity and placenta were 92% and 80%, respectively, indicating excellent performance ^(12)^. Furthermore, Lim et al. reported a deep-learning model to recognize the fetus on MRI ^(13)^.

Akazawa et al. developed a deep-learning model to predict severe hemorrhage (intraoperative blood loss > 2000 mL) based on MRI of patients with placenta previa, patient information, and blood test data. The predictive ability of this model was evaluated through comparison with the predictions of obstetricians and gynecologists. Of the 48 enrolled patients, 26 (54.2%) had blood loss ≥2000 mL and 22 (45.8%) had <2000 mL. The multimodal deep-learning model developed by obstetricians and gynecologists exhibited an accuracy of 0.68, an AUC of 0.74, and a median predictive ability of 0.61 ^(14)^. Zheng et al. developed a machine-learning model to predict placenta accreta based on MRI and clinical information, such as history of uterine surgery and placenta previa, with an AUC of 0.85 ^(15)^. Although MRI is relatively easy to perform in AI research as the subject is generally fixed, the sample size is smaller than that of CTG and echocardiography due to the lower frequency of examination. Thus, new algorithms with good accuracy even with small sample sizes are imperative.

### Others

With the new gestational diabetes mellitus (GDM) diagnostic criteria have made the diagnosis of GDM more rigorous, increasing the number of eligible patients and potentially causing financial and emotional concerns, Kurt et al. developed a model for identifying pregnant women at risk of GDM using deep learning to reduce unnecessary oral glucose tolerance tests for pregnant women with no GDM risk. Data were prospectively obtained from 489 pregnant women for the model development. A new decision support model for GDM was also developed, which exhibited 95% sensitivity, 99% specificity, and 98% AUC (95% CI: 0.95-1.00, *P* < 0.001) ^(16)^.

In addition, there have been some reports on the delivery mode model prediction. Using support vector machines, multilayer perceptrons, and random forest, Ramón et al. developed a machine-learning model for predicting three types of delivery: cesarean, normal, and instrumental. This model predicted the mode of delivery for 25038 deliveries using 48 variables (e.g., age, parity, time between rupture of membrane and labor, whether labor was induced, and whether a painless delivery occurred) as input layers. The accuracy of cesarean delivery prediction was >90%, and the need for instrumental delivery was predicted at 86% ^(17)^.

## Reproductive Medicine

AI research is also active in reproductive medicine. In Japan, assisted reproductive technology (ART) has been approved as an insured treatment in 2022. Therefore, AI research has the potential to become even more popular in the future. In ART, it is important to determine the quality of oocytes. Targosz et al. developed a model for classifying oocytes using deep learning from microscopic images. In this study, two types of deep neural networks were utilized: one for extracting specific regions of oocyte images and the other for classifying them. The accuracy of the model was 0.957, which is a good result ^(18)^. Furthermore, it is important to identify high-quality sperms in assisted reproductive medicine, and several AI studies have been conducted ^(19)(20)(21)(22)^ For example, Sato et al. developed an AI model for the morphological evaluation of sperms under a microscope. The sensitivity and positive predictive value for detecting abnormal and normal sperms were 0.881 and 0.853 and 0.794 and 0.689, respectively ^(22)^.

Several retrospective studies have employed AI to identify embryos with high implantation and pregnancy rates ^(23), (24), (25), (26)^. Khorsravi et al. developed a deep-learning model (STORK) for selecting high-quality embryos using a large collection of human embryo time-lapse images (approximately 50,000) from a large US fertility center. STORK exhibited an AUC >0.98, which was better than that of individual embryo cultures. Clinical data of 2182 embryos were utilized to generate a decision tree for determining embryo quality and patient age. The pregnancy rates varied from 13.8% (age > 41 years, poor quality) to 66.3% (age < 37 years, good quality) ^(25)^. Bormann et al. developed a deep-learning model to select good embryos from 97 clinical patient cohorts (742 embryos) using single-timepoint images of embryos collected 113 h after *in vitro* fertilization. The accuracy of the developed model was 90%, exceeding the ability of 15 trained embryo cultivators to identify good embryos ^(26)^.

The major cause of implantation failure and abortion is embryo aneuploidy, and preimplantation genetic testing for aneuploidy (PGTA) is one of the latest techniques for aneuploid embryo identification as it enables accurate analysis of chromosome numbers ^(27), (28)^. However, PGTA is an invasive and expensive test, and several AI-assisted ploidy prediction studies are currently underway ^(29), (30), (31), (32)^. Ma et al. retrospectively analyzed 3448 blastocysts from 979 time-lapse PGT cycles and used deep learning to develop a ploidy prediction model. The developed model exhibited an AUC of 0.612, which increased to 0.688 with the addition of clinical and embryological features ^(32)^. Thus, there are various reports on ART-related AI research. However, the accuracy is unsatisfactory, with few AI medical devices approved. Larger sample sizes and new deep-learning models are necessary to enhance clinical application.

As intrauterine adhesions and lesions, such as uterine polyps, can cause implantation failure, AI research has been conducted. Li et al. developed a deep-learning model for predicting infertility outcomes after hysteroscopic adhesiolysis. They utilized 4,922 hysteroscopic images of 555 cases of intrauterine adhesions after hysteroscopic adhesiolysis. The primary outcome was the ability to predict pregnancy within 1 year following adhesiolysis, reaching an AUC of 0.95 ^(33)^. Zhao et al. developed a deep-learning model for accurately detecting endometrial polyps for resection. In addition, a video adjacent-frame association algorithm was developed to address the problem of unstable polyp detection. The model was trained on a dataset of 11,839 images of 323 cases, and its diagnostic performance was confirmed using two datasets comprising 431 cases provided by two other hospitals. The results indicated that the sensitivity based on lesions reached 100% and 92.0% for the two test sets, respectively ^(34)^.

## Gynecologic Oncology

### Cervical cancer

Cervical cytology and human papillomavirus screening are important for the diagnosis of cervical cancer. Tao et al. developed an AI triage system to predict cervical intraepithelial neoplasia (CIN) 2+ lesions (atypical squamous cells of undetermined significance) using ASCUS cytology. The system achieved high sensitivity (92.9%; 95% CI: 75.0%-98.8%) and specificity (49.7%; 95% CI: 45.6%-53.8%) ^(35)^.

Colposcopy is employed to diagnose precancerous CIN and early-stage cervical cancer. The association between AI use and colposcopy is a frequently reported research area in obstetrics and gynecology ^(36), (37), ,(38)(39), (40), (41), (42), (43)^. Shinohara et al. developed a deep-learning model to segment CIN lesions before acetic acid treatment and yielded good results, with accuracy, precision, and F1 score of 0.894, 0.837, and 0.834, respectively ^(43)^. An AI-equipped colposcopy equipment has been used overseas. Kim et al. determined whether the diagnostic ability of four colposcopists (2 skilled and 2 inexperienced) with and without the Cerviray AI^Ⓡ^ system (AIDOT, Seoul, Korea) improved. Sensitivity and specificity improved when an AI system aimed at colposcopic lesion prediction and diagnosis was used ^(44)^. This study included 19,435 patients from six hospitals across China. The dataset included colposcopic images, clinical information, and pathology results (gold standard). The agreement between colposcopic pathology and the lesion site evaluated using CAIADS was higher than that interpreted by the colposcopists ^(45)^. Cho et al. developed a deep-learning model for diagnosing CIN1, CIN2, CIN3, and non-neoplasms using 1106 pathological images from 588 patients. The mean areas under the receiver operating characteristic curve for each class using EfficientNet-B7 were 0.996, 0.971, 0.956, and 0.990 for the non-neoplasm, CIN1, CIN2, and CIN3 groups, respectively ^(46)^.

Zhang et al. analyzed the clinical data and representative hematoxylin and eosin (HE)-stained pathological images of cervical cancer patients and developed a deep-learning model for predicting the 5-year survival. They retrospectively collected data from 238 patients with nonsurgical cervical cancer who had received chemoradiotherapy. After segmenting the HE-stained images into patches of 224 × 224 pixels, deep features were extracted. The AUC of the developed deep-learning model was 0.750 (95% CI 0.540-0.959) ^(47)^. Li et al. developed an AI model for diagnosing histological types of cervical cancer from 8496 histological images of 229 cervical samples (squamous cell carcinoma [SCC] of the cervix, n = 37; cervical adenocarcinoma, n = 8; nonmalignant cervical tissue, n = 184). The AI model distinguished SCC from adenocarcinoma ^(48)^. MRI can be employed to examine the local extent of cervical cancer, and there have been reports on deep learning in MRI for cervical cancer. Qian et al. developed a deep-learning model to predict normal-sized lymph node metastasis (LNM) from MRI of cervical cancer. The dataset utilized in this study included MRI data and patient information (age, tumor diameter, stage, apparent diffusion coefficient [ADC] maps, SCC value) of 169 patients with cervical cancer, and the LNM status was determined via histopathological examination. The accuracy of the developed diagnostic model reached an AUC of 0.890, and good results were obtained ^(49)^. Zhang et al. developed a deep-learning model for predicting pathological features that require adjuvant radiotherapy from MRI of early-stage cervical SCC. A deep-learning model developed from a dataset of MRI of 289 patients who underwent radical hysterectomy and pelvic lymph node dissection exhibited high accuracy and ability for predicting radiotherapy, as indicated by an AUC of 0.79 (95% CI: 0.67-0.90) in the test cohort ^(50)^.

### Endometrial cancer, uterine sarcoma

 There have been numerous reports of research on the diagnosis of endometrial cancer in MRI images using artificial intelligence. Urushibara et al. trained the CNN for predicting endometrial cancer on MRI of 204 endometrial cancer and 184 noncancerous lesions, reaching an AUC of 0.88-0.95 ^(51)^. In endometrial cancer, deep myometrial invasion is one of the risk factors for recurrence, and MRI is necessary for preoperative diagnosis, although it can be occasionally difficult to diagnose. Xiong et al. developed an AI evaluation model for the deep myometrial invasion of endometrial cancer using MRI of 75 patients with stage IA and 79 patients with stage 1B endometrial cancer. The model achieved an accuracy of 86.9%, sensitivity of 81.8%, and specificity of 91.7% ^(52)^. MRI can predict the histological type of endometrial cancer. Compared with other endometrial cancers, endometrial carcinosarcoma has a poorer prognosis. However, preoperative biopsy may contain errors as it has various components. Saida et al. developed a deep-learning model for predicting endometrial carcinosarcoma using MRI of 52 patients with uterine carcinosarcoma and 279 with endometrial carcinoma. The model exhibited the same or better diagnostic performance than experienced radiologists ^(53)^. Recently, immune checkpoint inhibitors have become key drugs in the treatment of endometrial cancer, and microsatellite instability (MSI) is a biomarker of this efficacy. A deep-learning model was developed using HE-stained samples of 529 patients with endometrial cancer in the Cancer Genome Atlas (TCGA), with an accuracy > 90% ^(54)^. In recent years, it has been proposed that uterine cancer (endometrioid cancer and serous cancer) can be classified into four molecular subtypes based on comprehensive genetic analysis of TCGA data: POLE type, MSI type, copy-number high type, and copy-number low type. Fremond et al. aimed to develop a deep-learning model for predicting four molecular subtypes of endometrial cancer from histopathological images and clinical data from a PORTEC randomized trial. The deep-learning model was able to perform molecular classification of endometrial cancer with high accuracy. In particular, it exhibited excellent performance in classifying POLE mutants and MSI types ^(55)^. In recent years, hysteroscopy has been employed not only for the diagnosis of benign diseases, such as uterine fibroids and endometrial polyps, but also for the diagnosis of endometrial cancer. Takahashi et al. aimed to develop an automatic diagnostic system for endometrial cancer using deep learning based on the hypothesis that hysteroscopy could be an important tool for the screening of endometrial cancer. In addition, they developed an original algorithm that achieved good accuracy even with a small number of cases. A total of 177 patients (normal endometrium, 60; hysteromyoma, 21; endometrial polyp, 60; endometrial atypical hyperplasia, 15; and endometrial cancer, 21) underwent hysteroscopy. The images were divided into two groups: malignant (atypical endometrial hyperplasia) and nonmalignant (normal endometrium, endometrial polyps, and fibroids). Deep learning for the prediction of the two groups achieved an accuracy > 90% ^(56)^. Uterine sarcoma is a soft tissue malignant tumor arising from the myometrium and has a poor prognosis. MRI is useful for the preoperative diagnosis of uterine fibroids and sarcomas. However, it is occasionally difficult to distinguish between degenerative fibroids and uterine sarcomas. Toyohara et al. developed a diagnostic model for uterine sarcomas using deep-learning analysis of MRI and postoperative pathological diagnoses. In a multi-institutional collaboration, we utilized preoperative MRI of 61 patients with uterine sarcoma and 200 with pathologically confirmed uterine fibroids following surgery. Deep-learning analysis was conducted using T1-, T2-, and diffusion-weighted images. Furthermore, we compared the accuracy of postoperative pathological diagnosis between the established model and radiologists (specialists). The accuracy rates of the developed AI model, residents, and specialists were 91.3%, 80.1%, and 88.3%, respectively; the results of our algorithm were comparable with those of radiologists ^(57)^. The annotation of the lesion site to develop an AI model for medical imaging is usually performed manually. Therefore, it becomes the rate-limiting step in research when a large amount of data is required. Toyohara et al. developed a system that automatically extracts the lesion site using MRI of uterine sarcoma and uterine fibroids and then automatically diagnoses the extracted image using AI. The accuracy, sensitivity, and specificity of the developed model were 92.44%, 92.25%, and 92.50%, respectively. The AI system developed in this study extracted lesion site data from MRI without human intervention and could diagnose uterine sarcomas with an accuracy comparable to that of radiologists. With further verification, the AI model could be used in diagnosing other diseases ^(58)^.

### Ovarian cancer

Ovarian cancer is the most fatal gynecological cancer. It has been the subject of many AI studies. The development of a deep-learning model for predicting malignancy and nonmalignancy from MRI findings has been reported ^(59), (60)^. Saida et al.’s study included 194 patients with pathologically confirmed ovarian cancer or borderline malignancy, as well as 271 patients with non-cancerous lesions. MRI analysis was performed using T2-weighted imaging (T2WI), diffusion-weighted imaging (DWI), apparent diffusion coefficient (ADC) maps, and fat-suppressed T1-weighted imaging (T1WI). The CNN model in this study was trained using 1,798 images from 146 cancer patients and 1,865 images from 219 non-cancer patients. The training was conducted separately for each image type. The performance of the CNN was evaluated using a test dataset consisting of 100 images of 48 malignant and 52 nonmalignant lesions. The developed CNN exhibited a performance equivalent to that of experienced radiologists in the diagnosis of ovarian cancer using MRI ^(59)^. Preoperative diagnosis of ovarian cancer and borderline malignancies of the ovary are essential for determining treatment strategies. However, the diagnosis is occasionally difficult. Wang et al. developed a deep-learning model to predict 102 borderline ovarian tumors and 89 ovarian cancers using T1- and T2-weighted images. The AUC was 0.87; accuracy, 83.7%; sensitivity, 75.0%; and specificity, 87.5%, exceeding the radiologist’s assessment (0.75%, 75.5%, 96.0%, and 54.2%, respectively; *P* < 0.001) ^(61)^. Ultrasonography is a minimally invasive, simple, and effective method for diagnosing ovarian cancer. Studies on ultrasound and AI in ovarian cancer have also been conducted ^(62), (63)^. Gao et al. developed an ovarian cancer detection model using multi-institutional ultrasound images of 3755 ovarian cancer patients and 101777 healthy individuals. Compared with radiologists, the developed model was more accurate in detecting ovarian cancer in the internal validation dataset (88.8% vs. 85.7%). The diagnostic assistance provided by the model improved the diagnostic accuracy, which reached 87.6%, and sensitivity of the radiologists ^(62)^. A study on pathological imaging and AI in ovarian cancer has also been reported ^(64), (65)^, in which a pathological image-based deep-learning classifier named PathoRiCH (Pathologic Risk Classifier for high-grade serous ovarian cancer) was able to predict platinum-based treatment response in high-grade serous ovarian cancer ^(64)^.

Thus, there are more reports on AI research in gynecological cancer than in other obstetrics and gynecology fields. The accuracy of molecular-subtype prediction and MSI prediction from pathological images in endometrial cancer is high. However, some studies have demonstrated low accuracy. Thus, larger sample sizes and novel algorithms with good accuracy even with a small number of cases are warranted.

## Problems with Medical AI Research

AI models rely on training data. If incomplete or biased data are utilized, the accuracy of the diagnosis and prediction decreases. Furthermore, when data are collected from multiple institutions to increase the number of data, domain shifts between institutions and medical devices where the training and test data do not match are likely to occur. Therefore, research on the development of good AI models with incomplete or biased data is important. In Japan, strict privacy protection is required in the handling, storage, and sharing of patient medical data. Unauthorized access and data leakage can lead to serious problems. AI-related laws and regulations have been revised and introduced in Japan and overseas; however, they remain incomplete. Many AI algorithms are referred to as “black boxes,” and it is difficult to understand how they achieve results. Furthermore, transparent and explainable AI decision processes are important for healthcare professionals and patients to improve trust. A method for visualizing this black box is to display a heat map using gradient-weighted class activation mapping. The introduction of AI may change roles and the required skill sets of healthcare professionals. Education and training in response to this change are necessary ^(66), (67)^. However, there is no consensus on the extent to which healthcare professionals need to learn and understand AI. In the future, it will be necessary to establish an educational curriculum for this purpose. AI will help healthcare professionals in making decisions by recognizing patterns and relationships in vast datasets. In healthcare settings, AI adoption is expected to automate repetitive tasks, enabling healthcare professionals to focus on more complex decisions and patient communication. AI will become part of the infrastructure that connects patients and providers regarding patient monitoring and preventive care delivery, allowing for a more continuous, personalized care ^(68)^.

## Currently Approved AI Medical Devices for Obstetrics and Gynecology and Future Prospects

The following are examples of currently approved AI medical devices in obstetrics and gynecology. Embryoscope+ is an AI-based system that monitors embryo development and evaluates its quality. It has been approved in Japan for improving the success rates of *in vitro* fertilization ^(69)^. DYSIS Ultra is an AI-powered colposcope approved by the US Food and Drug Administration and is CE-marked in Europe. Its special feature is that the AI automatically generates a DYSIS map based on the analysis results, showing abnormal areas in different colors. This provides visual support to the doctor when making a diagnosis (https://dysismedical.com/products/dysis-ultra/).

Prospects include diagnosis and treatment prediction models that integrate various modal data with AI. For example, the Cabinet Office project BRIDGE, which we are involved in, is building the first digital medical data bank in Japan that links medical, genome, medical imaging, and drug data of each patient. We aim to realize an AI-driven next-generation medical-care workflow and apply it to drug discovery and medical device development.

## Conclusion

AI research has been actively conducted in the fields of obstetrics and gynecology, and numerous reports have been published. However, not only technological advances but also legal, ethical, and social frameworks need to be established before these studies can be applied to clinical practice.

## Article Information

### Conflicts of Interest

None

### ORCID iD

Kenbun Sone: https://orcid.org/0000-0002-7218-6401

### Disclaimer

Yutaka Osuga is one of the Editors of JMA Journal and on the journal’s Editorial Staff. He was not involved in the editorial evaluation or decision to accept this article for publication at all.
